# Development of the scale of hygıene behavıors for nursıng students

**DOI:** 10.1186/s12874-015-0064-4

**Published:** 2015-08-21

**Authors:** Gulay Ipek Coban, Sonay Bilgin

**Affiliations:** Department of Fundamentals of Nursing, Ataturk University Faculty of Health Science, Erzurum, 25240 Turkey; Department of Public Health Nursing, Ataturk University Faculty of Health Science, Erzurum, 25240 Turkey

## Abstract

**Background:**

There is a need to have an appropriate instrument to measure the hygiene behaviors for nursing students. This study was carried out to develop a Hygiene Behavior Scale (HBS).

**Methods:**

The population of the study is composed of the students of students of nursing department. A total of 416 participants were included in this study. The students in the sampling group were asked to write a composition containing their feelings and thoughts about hygiene. These compositions were analysed and 87 items about positive and negative behaviors were determined. These items were presented to expert opinion and after necessary editions, reliability and validity analyses were conducted.

**Results:**

The resulting HBS consists of 25 items across the following three domains: Personal hygiene, handwashing technique and food-related hygiene . The final model in confirmatory factor analysis showed that this 25-item HBS indicated a good fit of the model. The value of the Cronbach’s a for the total scale was 0.90.

**Conclusions:**

The HBS is determined to be quite highly valid and reliable, sufficient measuring instrument to determine hygiene behaviors of nursing students.

**Electronic supplementary material:**

The online version of this article (doi:10.1186/s12874-015-0064-4) contains supplementary material, which is available to authorized users.

## Background

Hygiene is the key control measure to prevent hospital-acquired infections. Healthcare-associated infections (HAIs) result in excess deaths, length of hospital stay and healthcare costs [1–5]. With the aim to reduce healthcare-associated infections and the spread of antimicrobial resistance, the World Health Organization (WHO) World Alliance of Patient Safety launched the first Global Patient Safety Challenge [6] in October 2005 under the banner,‘Clean Care is Safer Care’.

Given the importance of hygiene behavior, we found it surprising that no widely available self-report measure to assess this behavior is available in Turkey. Consequently, we aimed to develop and test such a measure.

One major problem associated with studying hygiene behavior is how to measure it. Self-report, may be affected by a participant’s need to project socially desirable hygiene standards as with direct observation may be difficult. [7]. For hand hygiene behavior, measurement has relied on self-report, observation, and proxy measures (eg, illness rates, soap usage) [8]. As far as self-reporting hand hygiene instruments go, there are currently no validated measures, and those that are available tend to be group-specific (eg,nursing students). More broadly (ie, outside of hand hygiene), there appear to be no measures focusing primarily on hygiene behavior. Bulbul Maras et al. [9] were developed “Hand Washing Behavior Scale Terms of Planned Behavior Model” in Turkey. This scale is measure only hand washing behaviors. Kahveci and Demirtas [10] were developed “Cleaning and Hygiene Scale” that aim to measure perception of the Primary School Students about cleaning and hygiene, this scale can not be used for nursing students.

The role of the professional nurse in preventing hospital infections is significant. The hygiene practices of nursing students are an important area to examine because nursing students are the future work force and preregistration training provides the opportunity to address any factors leading to non-compliance with hygiene practices [5]. Lymer et al. [11] have suggested that nursing students are in an ideal position to promote effective hygiene as they can act as agents of change in practice by sharing good hygiene knowledge and behaviors with qualified staff.

Three specific aims guided this investigation:

1. To generate items for Hygiene Behavior Scale (HBS) in Turkey.
2. To evaluate the developed HBS for content, face and construct validity; internal consistency; test–retest reliability.
3. To develop and test psychometric properties of a new instrument for measuring the hygiene behaviors in Turkey

## Methods

### Design

This study was conducted in Erzurum, Turkey. The study phases were as follows: first, preparing item tool; second, content analysis by a panel of specialists; and third, psychometric testing (factor analysis, a reliability coefficient and inter-item correlations).

### Participants

The study was carried out in a faculty of health science between April 2013 and December 2013. The population of the study is composed of students of nursing department. The number of students of a faculty of health science nursing department 1-2-3th class were 446 and all of the students were included in the study. Among them 18 were unwilling to participate the study because of time shortage and 12 of them were not at the school on the days of making interviews. The study was completed with 416 students. Inclusion criteria were: able to comprehend and communicate using Turkish, no psychiatric history, self-reported absence of pain, willing to volunteer to complete the scale.

The authors searched for HBS-related instruments in the OVID databases, bibliographies and article references, and compiled a list of HBS items [12–15]. All participants ranged from 18 to 25 years (M = 21.33, SD = 2.17). The economic levels of all participants were:7.8 % high, 76.4 % middle, 15.8 % low, 148 males and 268 females. The educational levels of students’ parents were diverse (52.2 % primary school or less; 33.6 % high school; 14.2 % university) (Table 1). The compositions were analysed and 213 items about positive and negative behaviors were determined. The items that were explaining the same attitude were deleted and 87 items were taken for statistical analyses.

**Table 1:** Characteristics of students (*n* = 416)

Characteristics Mean ± SD	
Age (years), 21.33 ± 2.17	
	*n* (%)
Gender	
Male	148 (35.5)
Female	268 (64.5)
Economic status	
High	32 (7.8)
Middle	318 (76.4)
Low	66 (15.8)
Education levels of parents	
Primary scholl or less	217 (52.2)
High school	140 (33.6)
University	59 (14.2)

### Content validity

To test item clarity and content validity, the items were submitted to 10 nursing specialists and two sociologists who were informed of the measures and concepts involved. Each expert was asked to individually and independently evaluate and score each HBS item for its appropriateness (the item can be used in the HBS), representativeness (the item expresses a core concept of the HBS) and explicitness (the item is clearly stated and easy to understand) using a five-point Likert scale (1 = irrelevant and should be deleted; 2 = seemingly relevant but large-scale revision required; 3 = relevant but in need of small adjustments; and 4 = relevant, but needs rewording; 5 = relevant, clear and precise). Items with a mean score of 4.0 were retained. The decision to delete items scoring < 4.0 was made by the researchers based on the experts’ opinions. This pencil-paper scale takes around 7 min to complete. The respondent is asked to rate each item on a four-point Likert scale ranging from 1 for ‘always’ to 4 for ‘never’.

### Internal consistency

Item analysis was conducted to select items that were highly correlated with each other in each scale and to also reduce the number of items as much as possible, without decreasing internal consistency. Internal consistency might be a necessary condition for homogeneity or unidimensionality of a scale but, despite satisfying Cronbach’s a (40–80), it is nevertheless possible that the scale is not unidimensional [16]. The item–total correlation had to be > 0.30 and the value of Cronbach’s a should not decrease substantially when an item is dropped. Item deletion started with the item with the lowest internal correlation. When this item was deleted, internal consistency was re-estimated and the item with the next lowest item–total correlation was then deleted [17].

### Stability

The stability of the scale was established by measuring the test–retest reliability. In this study, the respondents were sent the same instrument after 2 weeks with the request to complete it again. Based on a code each respondent received, their data relating to the first and second measurement could be detected and matched. Then, by means of the interclass correlation coefficient, the test–retest reliability could be calculated.

### Construct validity

The data were analysed by means of factor analysis. To attain the best-fitting structure and correct number of factors, the following criteria were used: eigenvalues > 1.0, factor loadings > 0.40 and the so-called elbow criterion regarding the eigenvalues. Before conducting the factor analysis, the Kaiser–Meyer–Olkin measure of sampling adequacy (KMO) and Bartlett’s test were conducted to evaluate whether the sample was large enough to perform a satisfactory factor analysis. A KMO value > 0.5 indicates that the sample size is adequate for factor analysis.

The factor structure of the instrument was tested with explanatory factor analysis (EFA) and the factor loadings were calculated (Table 2) and confirmatory factor analysis (CFA). EFA can identify the factor structure for a set of variables based on data instead of theory. In contrast, CFA is generally based on a strong theoretical and empirical foundation that allows the investigator to specify a hypothesized factor structure in advance and then test it. Thus, CFA can determine how well the proposed model fits the data [18,19].

**Table 2:** Factor Loadings for the HBS(*n* = 416)

Items	Factor Loadings		
	PH	HWT	FRH
Upon getting home I take a shower	.872	4.756E-02	.248
Upon getting home I wash my hands	.847	5.899E-02	.214
I clean my teeth three times a day	.803	.298	.196
I brush my teeth during 2 or 3 min	.722	.275	.213
Before using the toilet, I wash my hands	.710	.248	.204
After using the toilet, I wash my hands	.625	.203	.195
After touching a pet or other animal, I wash my hands	.600	.194	.131
When I use a public toilet, I cover the seat with paper	.541	.195	.195
I don’t wear the same top or shirt two days in a row	.522	.192	.192
I don’t wear the same skirt or pants two days in a row	.501	.175	.175
I don’t wear the same underclothes two days in a row	.495	.132	.132
I don’t go without a wash, shower or bath two days in a row	.421	.121	.121
I don’t wear anyone’s (friend, brother or sister etc.) clothes	.399	.119	.119
When warm water is available, I wash my hands with warm water	.118	.778	−2.01E-02
I use antibacterial gel or wipes to clean my hands	7.665E-03	.682	.154
I wash my hands minimum in 1–2 min.	.204	.669	-.236
I wash my hands with soap	.202	.551	.147
After washing my hands, I dry my hands completely	.192	.503	.214
After washing my hands, I dry my hands with a disposable towel	.185	.444	.198
Before preparing food, I wash my hands	1.022E-02	.219	.749
Before eating food I wash my hands	.336	.205	.733
I wash fruit and vegetables before I eat them	.278	.187	.720
I don’t eat unpackaged foods	.203	.304	.674
I always look the expiration dates of foods in markets	.194	.142	.531
After handling raw foods and before handling cooked foods, I wash my hands	.128	.150	.302

*PH* Personal Hygiene, *HWT* Hand Washing Technique, *FRH* Food- Related Hygiene

### Ethical considerations

The study was approved by the Ataturk University Faculty of Health Science Etic Committee, and informed consent was obtained from each participant. Students were invited to participate in the study and were fully informed before verbal and written consent were obtained.

## Results

### Content validity

The scale, consisting of 87 items, was reviewed by the expert panel for its relevance and the phrasing of the items. Of the items, 37 which scored < 4.0 were deleted by the researchers based on experts’ opinions. This resulted in a final draft of 50 items.

### Internal consistency

The data were analysed with the statistical computer program SPSS/PC (IBM Corporation, New York, USA), and Cronbach’s a was calculated by means of the reliability option. This resulted in a Cronbach’s a of 0.90. Pearson’s product–moment correlation of the scale’s items ranged from a minimum value of 0.24 to a maximum value of 0.87. Thus, 25 of items were deleted from the scale. This resulted in a final draft of 25 items. Item total score correlations of the scale varied between 0.36 and 0.74 of the final scale of 25 items. The exclusion of no item increased the Cronbach's alpha coefficient and item total score correlations of all items were above 0.30 (Table 3)

**Table 3:** Item-total correlations

Items	Mean	Item-total correlations	Cronbach’s α if item deleted
1	2.13	.50	.90
2	2.31	.67	.90
3	1.60	.63	.90
4	1.29	.36	.90
5	2.50	.63	.90
6	1.83	.72	.90
7	3.55	.60	.90
8	2.23	.59	.90
9	1.95	.71	.90
10	2.76	.58	.91
11	2.56	.74	.90
12	2.61	.71	.90
13	2.98	.73	.90
14	2.05	.68	.90
15	1.93	.72	.90
16	2.13	.50	.90
17	2.32	.67	.90
18	1.68	.63	.90
19	1.29	.37	.90
20	3.50	.63	.90
21	1.84	.72	.90
22	3.55	.61	.90
23	2.29	.58	.90
24	1.95	.71	.90
25	2.78	.57	.90

### Stability

One hundred of the research population complied with the request to complete the scale for the second time after 2 weeks. The final 25-item version of the HBS examined the test–retest reliability for the total scale and subscales. The test–retest correlations for the total scale was *r* = 0.90, *P* < 0.001; each of the subscales ranged from0.98 to 0.99.

### Construct validity

#### Exploratory factor analysis

Before factor construction of the scale could be observed, the KMO measure of sampling adequacy tests and Bartlett’s Test of Sphericity were calculated. Analyses showed that the KMO was 0.921, indicating that the sample was large enough to perform a satisfactory factor analysis and that the sample size was sufficient for psychometric testing of a 25-item scale. The Bartlett’s test was c2 = 8735.46; it was found that the results of both tests were statistically significant at the level of *p* < 0.0001 and were satisfactory for factor analysis. By using SPSS/PC, a principal component analysis was completed. The principal component analysis showed three factors with an eigenvalue higher than one. The post-rotational variances of the factors were 15.35, 10.99 and 10.23 %, respectively. The three factors all together explained 37.52 % of the variance (Table 4). After the factor analysis, these three factors identified as conceptual:

**Table 4:** The results of the principal component factor analysis for HBS (*n* = 416)

Factor number	Eigenvalue	Percentiles of variance	Cumulative percentiles
1	11.64	15.35	15.35
2	4.09	10.99	26.01
3	2.13	10.23	37.52

Factor 1: personal hygiene

Factor 2: hand washing technique

Factor 3: food- related hygiene

### Confirmatory factor analysis

The correlated three-factor model was a better fit than the other models. All fits indicated that the three-factor model had a satisfactory goodness of fit (c2 = 2075.76; d.f. = 737; RMSEA = 0.06, *P* = 0.000). Thus, the results of cross-validation provided further evidence for the construct validity of the HBS with 25 items.

## Discussion

The purpose of this study was to develop and test psychometric properties of a new instrument for measuring hygiene behaviors of nursing students in Turkey. Investigators identified three factors from the EFA: Factor 1 identified general personal behaviors towards hygiene; Factor 2 is concerned about the handwashing technique; Factor 3 is related to the food- related hygiene. As Nunnally and Bernstein asserted, EFA should not be used to confirm factor structure because EFA is a datadriven method for exploring the factor structure of a set of variables when no theory guides the analysis [20]. 18 CFA, on the other hand, is a theory-driven method. Therefore, investigators used CFA to determine whether the hypothesized model identified from EFA fits the data. They then modified the model accordingly. The CFA results continued to support the three factors of the HBS. Although the initial fit indices did not provide full support for this model, a modified three-factor correlated model resulted in better-fit indices. Moreover, this modified model cross-validated data from the second sample, providing stronger support for construct validity of this newly constructed HBS. It appears that the subscales based on the three first-order factors could be scored individually. Yet, the correlated factors suggested the existence of a higher-order latent variable that subsumes all three factors, presumably hygiene behaviors. The homogeneity or unidimensionality of items is a major issue in assessing the psychometric properties of an instrument. The Cronbach’s a coefficients for the HBS total scale (0.90) and each of the three subscales (0.98–0.99) indicated good internal consistency for this newly constructed instrument. Overall, the results of test–retest analysis suggested that the HBS and three of its subscales were relatively stable over a 2-week period. This study provides evidence to support the content, face and construct validity as well as the internal consistency and retest reliability of the HBS in a Turkish population.

### Limitations

This scale should now be further evaluated with a larger sample size, in different regions in Turkey and with diverse populations. The HBS should be checked with variables that affect hygiene behaviors of students (Additional file 1).

## Conclusions

The HBS should be tested in other populations as there are differences in language, culture and health systems in the way of attitudes. Because the initial draft of this instrument was in Turkish, tests of the psychometric properties of the HBS in a population of Turkish speaking participants should be easier to conduct. The HBS developed in this study can be used to assess the nursing students’ hygiene behaviors. The authors also suggest that new studies can be carried out about the relationship between hygiene behaviors and prevention of hospital acquired infections.

## Additional file

Additional file 1:
**Hygiene Behaviors Scale (HBS).** (DOCX 15 kb)

## Abbreviations

HBS: Hygiene behavior scale
HAIs: Healthcare-associated infections
KMO: Kaiser–Meyer–Olkin test
EFA: Explanatory factor analysis
CFA: Confirmatory factor analysis
RMSEA: Root mean square error of approximation
