# Supplementary material for: Development of the scale of hygıene behavıors for nursıng students
Source: BMC Med Res Methodol. 2015 Aug 21;15:69. doi: 10.1186/s12874-015-0064-4 (PMC4546221; doi:10.1186/s12874-015-0064-4)
Supplement: Additional file 1: — Hygiene Behaviors Scale (HBS). (DOCX 15 kb) [file 12874_2015_64_MOESM1_ESM.docx]

**HYGIENE BEHAVIORS SCALE (HBS)**

| **SCALE ITEMS** | **Always** | **Usually** | **Occasionally** | **Never** |
| --- | --- | --- | --- | --- |
| **PERSONAL HYGIENE** |  |  |  |  |
| Upon getting home I take a shower |  |  |  |  |
| Upon getting home I wash my hands |  |  |  |  |
| I clean my teeth three times a day |  |  |  |  |
| I brush my teeth during 2 or 3 minutes |  |  |  |  |
| Before using the toilet, I wash my hands |  |  |  |  |
| After using the toilet, I wash my hands |  |  |  |  |
| After touching a pet or other animal, I wash my hands |  |  |  |  |
| When I use a public toilet, I cover the seat with paper |  |  |  |  |
| I don’t wear the same top or shirt two days in a row |  |  |  |  |
| I don’t wear the same skirt or pants two days in a row |  |  |  |  |
| I don’t wear the same underclothes two days in a row |  |  |  |  |
| I don’t go without a wash, shower or bath two days in a row |  |  |  |  |
| I don’t wear anyone’s (friend, brother or sister etc.) clothes |  |  |  |  |
| **HAND WASHING TECHNIQUE** |  |  |  |  |
| When warm water is available, I wash my hands with warm water |  |  |  |  |
| I use antibacterial gel or wipes to clean my hands |  |  |  |  |
| I wash my hands minimum in 1-2 minutes. |  |  |  |  |
| I wash my hands with soap |  |  |  |  |
| After washing my hands, I dry my hands completely |  |  |  |  |
| After washing my hands, I dry my hands with a disposable towel |  |  |  |  |
| **FOOD- RELATED HYGIENE** |  |  |  |  |
| Before preparing food, I wash my hands |  |  |  |  |
| Before eating food I wash my hands |  |  |  |  |
| I wash fruit and vegetables before I eat them |  |  |  |  |
| I don’t eat unpackaged foods |  |  |  |  |
| I always look the expiration dates of foods in markets |  |  |  |  |
| After handling raw foods and before handling cooked foods, I wash my hands |  |  |  |  |
